# First report of two rapid-onset fatal infections caused by a newly emerging hypervirulent *K. Pneumonia* ST86 strain of serotype K2 in China

**DOI:** 10.3389/fmicb.2015.00721

**Published:** 2015-07-21

**Authors:** Yibo Zhang, Jingyong Sun, Chenrong Mi, Wenhui Li, Shengyuan Zhao, Qun Wang, Dake Shi, Luo Liu, Bingyu Ding, Yung-Fu Chang, Hongxiong Guo, XiaoKui Guo, Qingtian Li, Yongzhang Zhu

**Affiliations:** ^1^Department of Hospital Infection Control, Ruijin Hospital, Shanghai Jiao Tong University School of MedicineShanghai, China; ^2^Department of Microbiology, Ruijin Hospital, Shanghai Jiao Tong University, School of MedicineShanghai, China; ^3^Department of Laboratory Medicine, Ruijin Hospital, Shanghai Jiao Tong University School of MedicineShanghai, China; ^4^Department of Population Medicine and Diagnostic Sciences, College of Veterinary Medicine, Cornell UniversityIthaca, NY, USA; ^5^Department of STD and AIDS Control and Prevention, Jiangsu Provincial Center for Disease Control and PreventionNanjing, China; ^6^Department of Immunology and Microbiology, Institutes of Medical Sciences, Shanghai Jiao Tong University School of MedicineShanghai, China

**Keywords:** hypervirulent *Klebsiella pneumoniae* (hvKP), pyogenic liver abscess (PLA), ST86, virulence factors (VFs), iron-uptake system (*kfu*)

## Abstract

Here, we present the first report of one suspected dead case and two confirmed rapid-onset fatal infections caused by a newly emerging hypervirulent *Klebsiella pneumoniae* ST86 strain of serotype K2. The three cases occurred in a surgery ward during 2013 in Shanghai, China. A combination of multilocus sequence typing, pulsed-field gel electrophoresis, phenotypic and PCR tests for detecting virulence factors (VFs) was used to identify the isolates as K2 ST86 strains with common VFs, including Aerobactin and *rmpA*. Furthermore, the two K2 ST86 strains additionally harbored a distinct VF *kfu* (responsible for iron uptake system), which commonly existed in invasive K1 strains only. Thus, the unusual presence of both K1 and K2 VFs in the lethal ST86 strain might further enhance its hypervirulence and cause rapid onset of a life-threatening infection. Nevertheless, despite the administration of a combined antibiotic treatment, these three patients all died within 24 h of acute onset, thereby highlighting that the importance of early diagnosis to determine whether the ST86 strains harbor key K2 VF and unusual K1 *kfu* and whether patients should receive a timely and targeted antibiotic therapy to prevent ST86 induced fatal pneumonia. Finally, even though these patients are clinically improved, keeping on with oral antibiotic treatment for additional 2–3 weeks will be also vital for successfully preventing hvKP reinfection or relapse.

## Introduction

**FIGURE 1:**
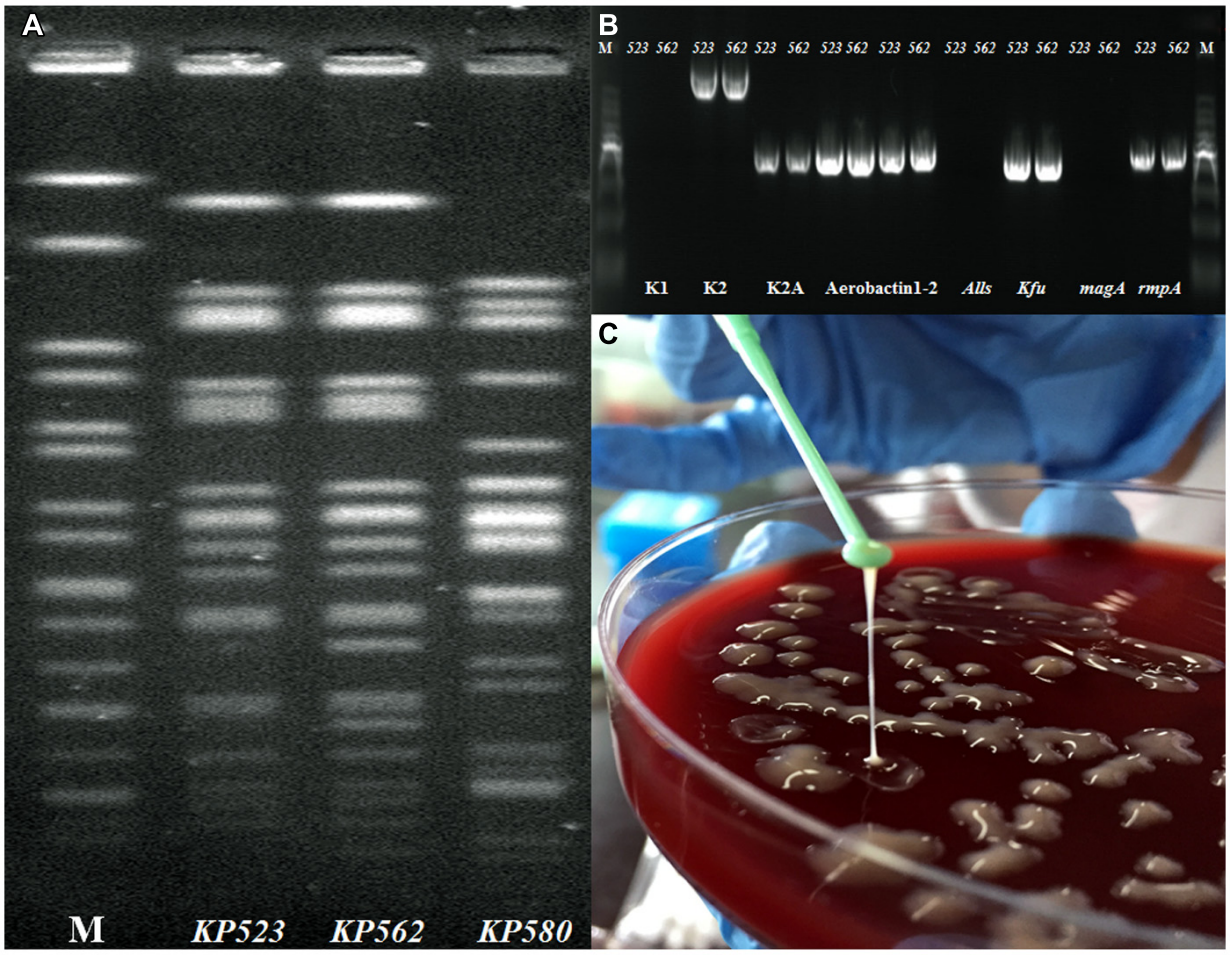
**The clinical and epidemiological characteristics of the three cases in this study**.

Here, we report that one suspected dead case and two confirmed, rapid onset and fatal infections caused by a *Klebsiella pneumoniae* ST86 strain occurred in a three-bed surgery ward of a university hospital with 1,800 beds and 40,000 annual admissions in Shanghai, China in 2013 (See **Figure 1**).

The first suspected patient, a 64-year-old male, after resuscitation from acute recurrent myocardial infarction, was sent to the surgery ward at 3 PM, on May 31th. In addition, the patient has the following diseases: coronary atherosclerotic heart disease, cerebral infarction, hypertension, type 2 diabete, and hyperlipoidemia. Since June 1st, the patient was initially treated empirically with moxifloxacin (400 mg daily) and gradually improved. At that time, the WBC count was 9.10 × 10^9^/L with 64.3% granulocytes and 20.7% lymphocytes. However, on June 4th, his WBC count increased to 9.59 × 10^9^/L with unusual 82.0% granulocytes and 8.1% lymphocytes, accompanied by a high temperature, breathing difficulty, and brick red foamy blood sputum, and followed by respiratory distress syndrome. Immediately, the patient was transferred into an intensive care unit (ICU) and died at 4 AM, on June 5th. Since samples were not collected as a result of the patient’s sudden death, this case was listed as a suspected case and potential source of infection only.

The second patient, a 72-year-old male, was admitted due to chordae tendineae of mitral valve and mitral incompetence on May 16th. At the beginning of admission, the patient showed clear breathing sounds, but no cough, expectoration, and wheezing sound. On May 29th, the patient recovered well in the ICU and was transferred to the same surgery ward. Until August 4th, the chest wound of the patient still didn’t heal well and followed by the exudates containing plenty of thick, tan-black colored secretion. The WBC count was 12.91 × 10^9^/L with 85.9% granulocytes and 7.9% lymphocytes. However, microbiological culture of tan-black colored secretion was negative. At 1 AM on June 8th, the patient’s body temperature suddenly increased to 39.2°C, accompanied by chest tightness, shortness of breath, coarse breath sounds, brick red foamy blood sputum, expectoration and wheezing as well as coarse rales. At that time, the WBC count decreased to 8.48 × 10^9^/L with 91.0% granulocytes and 4.7% lymphocytes. Subsequently, the patient was transferred into the ICU and immediately treated with linezolid and cefoperazone/sulbactam. However, his condition deteriorated late at night on June 8th and he eventually died from multiple organ failure caused by severe pneumonia and septic shock at 1:30 AM, June 9th. A routine microbiological culture of blood sample remained negative. However, a *K. pneumoniae* strain *Kp523* from a sputum sample was isolated.

The third patient, a 74-year-old male, having a long disease history of ischemic cardiomyopathy and old myocardial infarction, was the first one admitted to the ward on April 18th, because of the unsuccessful percutaneous coronary intervention, and daily treated with moxifloxacin (400 mg daily). However, on the night of May 25th, some unexpected symptoms, including chest tightness and shortness of breath, suddenly appeared for large effusions in right pleural and resulted in a restrictive ventilatory dysfunction. And the WBC count was 15.8 × 10^9^/L with 93.8% granulocytes and 3.8% lymphocytes. After discharging more than 1,100 ml dark-red colored effusions in right pleural, the patient condition obviously improved. And microbiological culture of the pleural effusion sample was still negative. Until June 3rd, the patient had almost completely recovered with most parameters of routine blood tests being near normal (6.3 × 10^9^/L WBC with 71.3% granulocytes and 16.0% lymphocytes). Thus antibiotic treatment was stopped. Unexpectedly, at 10:40 PM, on June 8th, the patient experienced acute pneumonia including high temperature (39.0°C), red foamy bloody sputum, breathing difficulty and low oxygen saturation, immediately followed by respiratory distress syndrome. The patient was rapidly transferred to the ICU. At 9 PM on June 9th, despite combination therapy of ganciclovir, imipenem/cilastatin, tigecycline, and hemofiltration, the patient deteriorated and finally died of multiple organ failure caused by severe pneumonia and septic shock at 11:45 PM, June 10th. Subsequent routine microbiological culture of blood sample successfully isolated *K. pneumoniae* strain named *Kp562*.

**FIGURE 2:**
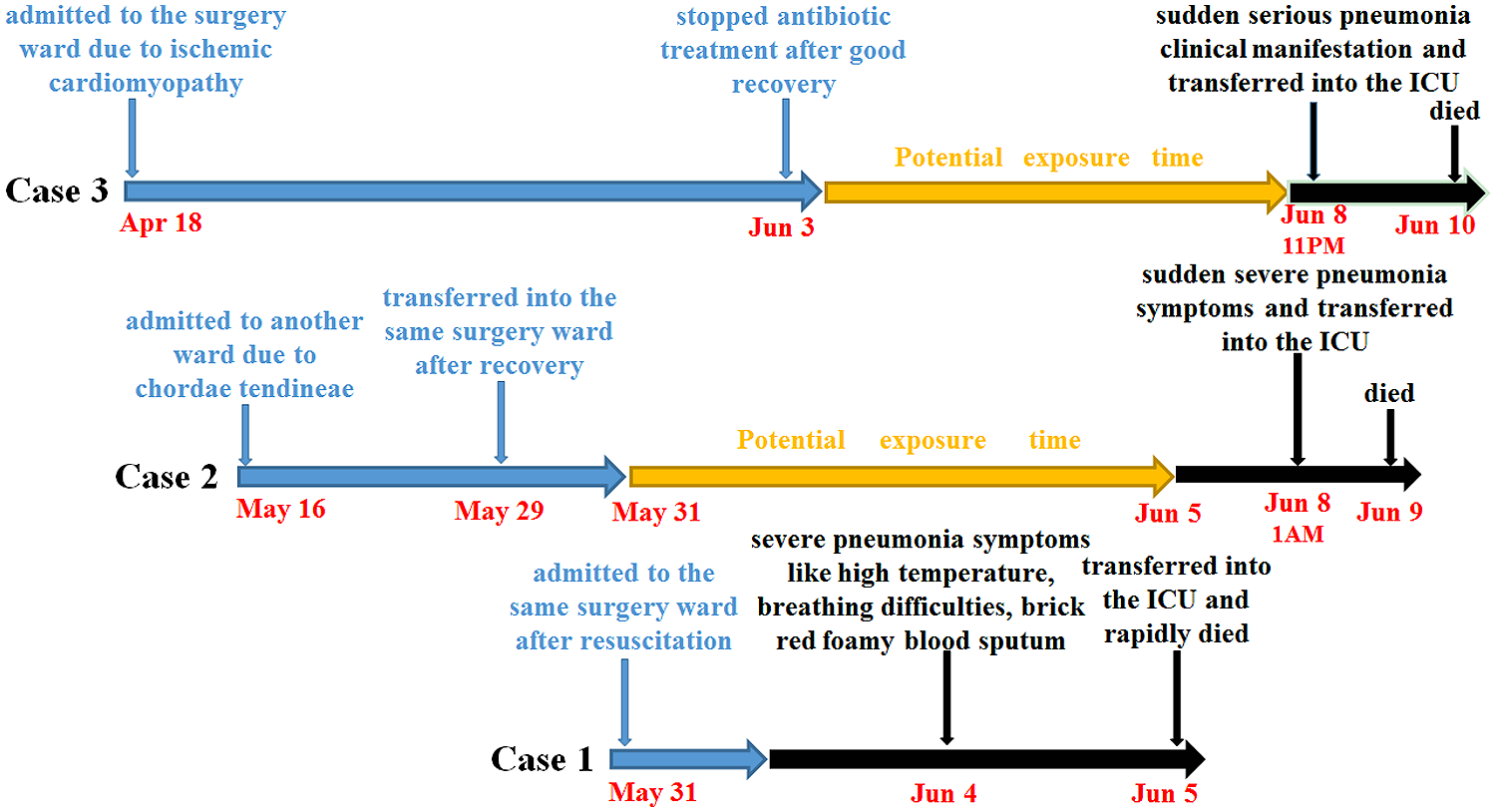
**(A–C)** Phenotypic and molecular characteristics identification of *KP523* and *KP562*. **(A)** Genomic DNA of *KP523*/*KP562* strains and another clinical strain *KP580* as control were digested using *XbaI* enzyme and subjected to PFGE; **(B)** PCR genotyping determining the presence or absence of the specific *wzx* gene for capsule serovar K1, K2, Aerobactin, *kfu, alls, magA*, and *rmpA* gene; **(C)** Positive hypermucoviscosity phenotype of *Kp523/562* by string test.

The protocol for this study was approved by Ethics Committee of Ruijin Hospital, Shanghai JiaoTong University School of Medicine. The combination of routine PFGE and MLST typing revealed that the two isolates shared highly similar PFGE profiles as *K. pneumoniae* ST86 (**Figure 2A**). Via PCR targeting *wzx* gene of capsule serovar K1, K2,(Yu et al., 2008) and aerobactin, *allS, kfu, magA*, and *rmpA* (Lin et al., 2014). The detailed PCR conditions listed in Supplementary Table S1. The two strains were confirmed as serotype K2 and Aerobactin-*kfu-rmpA* positive but *magA-allS* negative (**Figure 2B**). The two isolates grew as positive hypermucoviscous phenotype (HV) with a mucoviscous string > 5 mm in length from the colony on the blood agar plate (**Figure 2C**). Moreover, except nitrofurantoin and ampicillin, the two isolates exhibited susceptibility to the other 16 antimicrobial agents including ampicillin/sulbactam, piperacillin/tazobactam, cefazolin, cefotetan, ceftazidime, ceftriaxone, cefepime, aztreonam, ertapenem, imipenem, amikacin, gentamicin, tobramycin, ciprofloxacin, levofloxacin, and trimethoprim/sulfamethoxazole using Vitek 2 automated system (BioMérieux, St Louis, MO, USA) according the Clinical and Laboratory Studies Institute guidelines (CLSI2013).

## Background

During the previous decades, infections due to *K. pneumoniae* have primarily been attributed to classic *K. pneumoniae* (cKP) strains. However, since the first hypervirulent *K. pneumoniae* (hvKP) strain causing distinct pyogenic liver abscess (PLA) were reported in Taiwan in 1986, the new phenotypic hvKP strains have been increasingly common worldwide (Shon et al., 2013). Compared to the cKP strains, the unique clinical features of the hvKP strains are their ability to cause life-threatening community-acquired invasive infections (Shon et al., 2013). The invasive nature of the hvKP strains has been associated with multiple virulence factors (VFs), including K2-specific regulator of mucoid phenotype A gene (*rmpA*), and K1-specific mucoviscosity-associated gene A (*magA*), iron-uptake system (*kfu*) and HTH-type transcriptional activator (*allS*), (Yu et al., 2008).

In 2011, one report in France indicated that two hypervirulent K2 ST86 strains were more virulent than those of K1 ST23 strains (Decre et al., 2011). Actually, in 1991, the first ST86 strain-CG43 causing severe bacteremia and PLA was reported in Taiwan (Shon et al., 2013). Recently, two hypervirulent K2 ST86 strains have been reported including strain hvKP1 isolated from a 24-year-old Vietnamese male in New York, which caused a unique PLA accompanied by a metastatic spread to the spleen (Russo and Gill, 2013), while another strain, isolated in South Korea, caused severe bacteraemic infection (Jung et al., 2013). Currently, ST86 isolated from five PLA cases throughout Taiwan, Hong Kong, and Singapore have been reported (Lin et al., 2014). Here, this is the first report of hvKP ST86 strains causing one suspected and two confirmed fatal cases in one surgery ward in China.

## Discussion

So far, compared to major ST23 of K1, not much has been studies about ST86 strains. In the present study, we have experimentally confirmed the presence of the pivotal VFs closely associated with PLA or hypermucoviscosity phenotype. In the present study, the more distinct difference of the ST86 strains than other K2 strains is the additional presence of *kfu* gene exclusively in our strains. To date, most reported ST86 strains have possessed a HV, *rmpA*, and PLA phenotype but no *kfu*. For example, two recently published studies described that all of the seven hvKP ST86 strains from France, USA, and Taiwan and 11 ST86 strains from Hong Kong, Singapore, and Taiwan, harbored no *kfu* gene (Suzanne et al., 2013; Lin et al., 2014). However, the two *Kp523/562* strains exhibited HV and unique K2-Aerobactin*-kfu-rmpA* positive phenotypes, yet no PLA. This may be associated with the rapid, acute onset of disease caused by hypervirulence of ST86 strains; the patients were dead before PLA occurred. Furthermore, the HV-Aerobactin-*rmpA* positive strains of *K. pneumoniae* had previously been shown to be highly lethal to mice and were more virulent than HV-Aerobactin-*rmpA* negative strains (Yu et al., 2008). It was well-known that the K1 PLA strains carried *kfu* gene involved in human liver abscesses and metastatic spread infections, though this was generally absent in K2 strains; probably making the K1 strains significantly more prevalent than K2 (Yu et al., 2008). Therefore, the acquisition of *kfu* gene by the *Kp523/562* strains might further enhance their hypervirulence (Ito et al., 2015). In fact, it was recently found that certain K2 strains from other minor ST types, like ST375 and ST380, also possessed the *kfu* gene, reflecting that the presence of *kfu* gene might be closely associated with different genetic background or geographic origin among serotype K2 strains (Lin et al., 2014). It is unknown how K2 strains might obtain the *kfu* gene from K1 strains. Although virulence plasmid acquisition is one of major mechanisms acquiring new gene for hvKP strains, however, as a chromosomal virulence gene, the acquisition of *kfu* gene in K2 strains might occur via horizontal gene transfer mediated by bacteriophage or transposons. Whole genome sequencing of the two strains and comprehensively comparing them with other hvKP strains to further investigate the detailed mechanism of the acquirement of *kfu* in K2 ST86 strain is warrant in the near future.

Another important clinical characteristic of the hvKP ST86 strains is the high mortality rates likely due to their increased hypervirulence. Based on these findings above, among all of the 12 ST86-infected cases with detailed clinical information, ST86 strains totally caused five fatal infections, seven severe PLA, and one bacteraemic infection. It seems that mortality rate (41.6%, 5/12) caused by ST86 was obviously higher than that of other hypermucoviscositic strains, such as 7.3% in Canada, (Peirano et al., 2013), 3.0 and 31% in Taiwan (Ko et al., 2002; Ku et al., 2008). The significant mortality rate might be primary due to availability of very limited data reporting the prevalence and characteristics of ST86 worldwide.

On the other hand, despite the high prevalence of ST86 accounting for 46% PLA cases in other Asia countries/regions (Lin et al., 2014), there have only been two recent reports which concluded that only two out of 84 PLA-causing strains were ST86 strains in China. This suggests that both severe onset and rapid death were caused by its hypervirulence without PLA and that the prevalence of ST86 infection in China could be greatly underestimated (Li et al., 2014; Liu et al., 2014).

## Concluding Remarks

In this study, for the first time, unusual VF profiles correlating with the hypervirulence of ST86 strain causing two rapid-onset fatal infections were identified. However, regarding very little data of ST86 presently available, thus further molecular and epidemiological characterization studies should be performed to reveal the distribution and clinical importance of the unusual virulence factor profiles of hvKP ST86 strains originating from different geographic regions or countries outside Asia. Based on our finding, despite antibiotic therapy after acute onset, all of the two confirmed and one suspected patients rapidly deteriorated and eventually died. Therefore, combined application of MLST and VF detection implemented immediately to confirm the presence of hvKP ST86 strains should be crucially important for the doctor to early treat the patients with antibiotics if they carry the ST86 strains with Aerobactin-*kfu*-*rmpA* phenotype, even they don’t have any clinical signs of this disease at that time. Moreover, when these patients have been clinically improved, keeping on with oral antibiotic treatment for additional 2–3 weeks will be also vital for successfully preventing reinfection or relapse of hvKP strains.

## Conflict of Interest Statement

The authors declare that the research was conducted in the absence of any commercial or financial relationships that could be construed as a potential conflict of interest.
