# Supplementary material for: First report of two rapid-onset fatal infections caused by a newly emerging hypervirulent K. Pneumonia ST86 strain of serotype K2 in China
Source: Front Microbiol. 2015 Jul 21;6:721. doi: 10.3389/fmicb.2015.00721 (PMC4508851; doi:10.3389/fmicb.2015.00721)
Supplement: Supplementary file 1 [file Presentation_1.PPTX]

## Slide 1
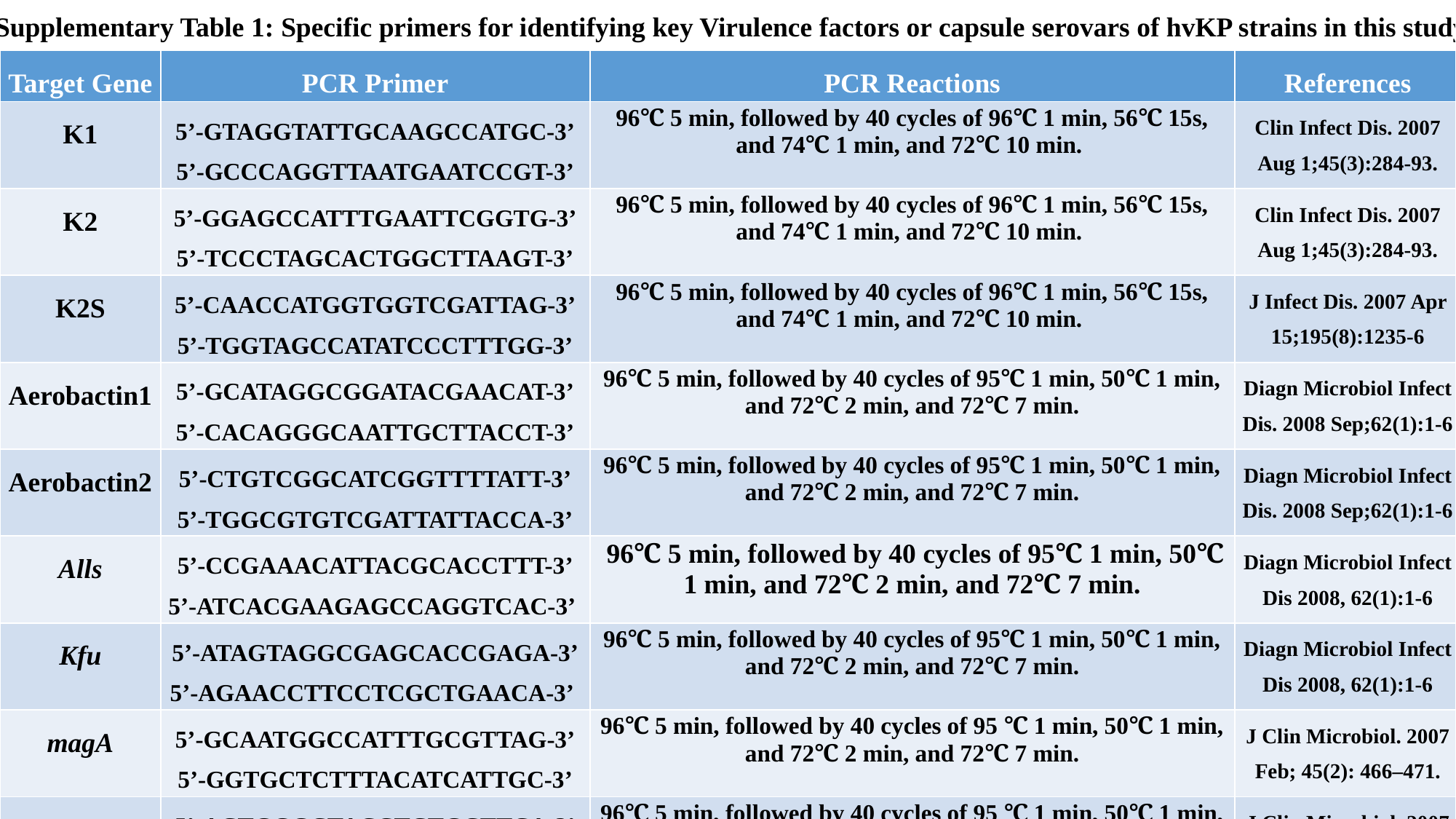

Supplementary Table 1: Specific primers for identifying key Virulence factors or capsule serovars of hvKP strains in this study
| Target Gene | PCR Primer | PCR Reactions | References |
| --- | --- | --- | --- |
| K1 | 5’-GTAGGTATTGCAAGCCATGC-3’ 5’-GCCCAGGTTAATGAATCCGT-3’ | 96℃ 5 min, followed by 40 cycles of 96℃ 1 min, 56℃ 15s, and 74℃ 1 min, and 72℃ 10 min. | Clin Infect Dis. 2007 Aug 1;45(3):284-93. |
| K2 | 5’-GGAGCCATTTGAATTCGGTG-3’ 5’-TCCCTAGCACTGGCTTAAGT-3’ | 96℃ 5 min, followed by 40 cycles of 96℃ 1 min, 56℃ 15s, and 74℃ 1 min, and 72℃ 10 min. | Clin Infect Dis. 2007 Aug 1;45(3):284-93. |
| K2S | 5’-CAACCATGGTGGTCGATTAG-3’ 5’-TGGTAGCCATATCCCTTTGG-3’ | 96℃ 5 min, followed by 40 cycles of 96℃ 1 min, 56℃ 15s, and 74℃ 1 min, and 72℃ 10 min. | J Infect Dis. 2007 Apr 15;195(8):1235-6 |
| Aerobactin1 | 5’-GCATAGGCGGATACGAACAT-3’ 5’-CACAGGGCAATTGCTTACCT-3’ | 96℃ 5 min, followed by 40 cycles of 95℃ 1 min, 50℃ 1 min, and 72℃ 2 min, and 72℃ 7 min. | Diagn Microbiol Infect Dis. 2008 Sep;62(1):1-6 |
| Aerobactin2 | 5’-CTGTCGGCATCGGTTTTATT-3’ 5’-TGGCGTGTCGATTATTACCA-3’ | 96℃ 5 min, followed by 40 cycles of 95℃ 1 min, 50℃ 1 min, and 72℃ 2 min, and 72℃ 7 min. | Diagn Microbiol Infect Dis. 2008 Sep;62(1):1-6 |
| Alls | 5’-CCGAAACATTACGCACCTTT-3’ 5’-ATCACGAAGAGCCAGGTCAC-3’ | 96℃ 5 min, followed by 40 cycles of 95℃ 1 min, 50℃ 1 min, and 72℃ 2 min, and 72℃ 7 min. | Diagn Microbiol Infect Dis 2008, 62(1):1-6 |
| Kfu | 5’-ATAGTAGGCGAGCACCGAGA-3’ 5’-AGAACCTTCCTCGCTGAACA-3’ | 96℃ 5 min, followed by 40 cycles of 95℃ 1 min, 50℃ 1 min, and 72℃ 2 min, and 72℃ 7 min. | Diagn Microbiol Infect Dis 2008, 62(1):1-6 |
| magA | 5’-GCAATGGCCATTTGCGTTAG-3’ 5’-GGTGCTCTTTACATCATTGC-3’ | 96℃ 5 min, followed by 40 cycles of 95 ℃ 1 min, 50℃ 1 min, and 72℃ 2 min, and 72℃ 7 min. | J Clin Microbiol. 2007 Feb; 45(2): 466–471. |
| rmpA | 5’-ACTGGGCTACCTCTGCTTCA-3’ 5’-CTTGCATGAGCCATCTTTCA-3’ | 96℃ 5 min, followed by 40 cycles of 95 ℃ 1 min, 50℃ 1 min, and 72℃ 2 min, and 72℃ 7 min. | J Clin Microbiol. 2007 Feb; 45(2): 466–471. |
Specific primers used for amplification of the target alleles of Klebsiella pneumoniae.
